# Beliefs about Binge Eating: Psychometric Properties of the Eating Beliefs Questionnaire (EBQ-18) in Eating Disorder, Obese, and Community Samples

**DOI:** 10.3390/nu10091306

**Published:** 2018-09-14

**Authors:** Amy L. Burton, Deborah Mitchison, Phillipa Hay, Brooke Donnelly, Christopher Thornton, Janice Russell, Jessica Swinbourne, Christopher Basten, Mandy Goldstein, Stephen Touyz, Maree J. Abbott

**Affiliations:** 1 School of Psychology, The University of Sydney, Sydney, NSW 2006, Australia; amy.burton@sydney.edu.au (A.L.B.); brooke.donnelly@health.nsw.gov.au (B.D.); janice.russell@sydney.edu.au (J.R.); drjessicaswinbourne@gmail.com (J.S.); stephen.touyz@sydney.edu.au (S.T.); maree.abbott@sydney.edu.au (M.J.A.); 2 Centre for Emotional Health, Department of Psychology, Macquarie University, North Ryde, NSW 2109, Australia; chrisb@bastenpsychology.com.au; 3 School of Medicine, Western Sydney University, Campbelltown, NSW 2560, Australia; p.hay@westernsydney.edu.au; 4 The Peter Beumont Eating Disorder Service, Royal Prince Alfred Hospital, Sydney, NSW 2006, Australia; 5 The Redleaf Practice, Wahroonga, NSW 2076, Australia; chris.thornton@theredleafpractice.com; 6 Mandy Goldstein Psychology, Bondi Junction, NSW 2022, Australia; mandy.goldstein@mgpsych.com

**Keywords:** binge eating, questionnaire, psychometric, eating disorders, obesity

## Abstract

Binge eating is a core diagnostic feature of bulimia nervosa, binge eating disorder, anorexia nervosa binge/purge type, and is a common feature of “other specified” and “unspecified” feeding and eating disorders. It has been suggested that specific metacognitive beliefs about food, eating, and binge eating may play a key role in the maintenance of binge eating behaviour. The Eating Beliefs Questionnaire (EBQ-18) provides a brief self-report assessment tool measuring three types of metacognitive beliefs: negative, positive, and permissive beliefs about food and eating. This study aimed to build on past research by validating the factor structure and psychometric properties of the EBQ-18 using both a clinical and non-clinical sample. A sample of 688 participants (*n* = 498 non-clinical participants, *n* = 161 participants seeking treatment for an eating disorder*,* and *n* = 29 participants seeking treatment for obesity) completed a battery of questionnaires, including the EBQ-18 and other measures of eating disorder symptoms and relevant constructs. A subset of 100 non-clinical participants completed the test battery again after an interval of two-weeks, and 38 clinical participants completed the EBQ-18 before and after receiving psychological treatment for their eating disorder. A confirmatory factor analysis (CFA) was conducted and psychometric properties of this measure were assessed. The results of this study provide support for the three-factor model of the EBQ-18. In addition, the EBQ-18 was found to be a valid and reliable measure, with excellent internal consistency, good test-retest reliability in the non-clinical sample, and also demonstrated evidence of sensitivity to treatment in clinical samples with binge eating pathology. Receiver operating characteristic (ROC) curve analyses were used to identify optimal cut-off scores for the EBQ-18. This study provides valuable information about the utility of the EBQ-18 as a measure for use in both clinical and research settings.

## 1. Introduction

Recurrent binge eating is a core diagnostic feature of binge eating disorder (BED), bulimia nervosa (BN), anorexia nervosa binge-purge type (AN-BP), and it is a common feature of “other specified” and “unspecified” feeding and eating disorders (OSFED; UFED [1]). A binge eating episode is defined as eating an objectively large amount of food in a discrete time period, accompanied by a sense of loss of control [1]. In BED, and often in other eating disorders, binge eating episodes are paired with feelings of guilt, disgust, marked distress, and/or low mood. To meet criteria for BED or BN, individuals need to have been engaging in binge eating episodes one or more times per week for a period of at least three months [1]. Population studies from the USA and Australia have found prevalence of approximately 1–1.5% for BN, 1.5–1.6% for BED, 0.4–0.5% for Anorexia Nervosa (AN), and 3.2% for OSFED [1,2]. The prevalence of recurrent binge eating in the general community has been found to range between 7.2% to 13%, with reports indicating that this prevalence is increasing over time [3,4]. This is a health behaviour of significant public interest due to its association with obesity [5], eating disorders [2] and other significant social, physical, and mental health concerns [6,7,8]. Known comorbidities of binge eating include anxiety, depression, substance abuse, chronic pain, diabetes, and obesity [9,10]. Furthermore, the presence of binge eating has been found to complicate obesity management and negatively impact the efficacy of obesity treatments [11,12,13].

In an attempt to understand the complex eating disorder symptom of binge eating better, a number of psychological models for BN and binge eating have been proposed in the literature, with many focusing on the role of restrained eating practices, poor self-esteem, poor distress tolerance, an overvaluation of body weight and shape, and specific unhelpful beliefs about binge eating (for a review of psychological models of binge eating see Burton & Abbott, 2017 [14]). In order to develop and evaluate effective psychological treatments for binge eating, it is important to be able to reliably measure the features underlying the development and maintenance of binge eating. Validated measures exist for the measurement of poor self-esteem (e.g., Rosenberg’s Self-Esteem Scale), poor distress tolerance (e.g., Difficulty with Emotional Regulation Scale and Distress Tolerance Scale), as well as the frequency of binge eating itself, restrained eating, the overvaluation of body shape and weight (e.g., Eating Disorders Examination), and others features that are relevant to binge eating [15]. However, to our knowledge there are only three measures available for the assessment of beliefs about binge eating predicted to maintain binge eating in individuals with eating disorders: the Eating Disorders Thought Questionnaire, Eating Disorders Core Belief Questionnaire, and the Eating Beliefs Questionnaire (EBQ). Of these, only the EBQ has been validated with a clinical sample [15].

The eighteen-item self-report Eating Beliefs Questionnaire (EBQ-18) consists of three subscales (negative, positive, and permissive beliefs about eating) with each subscale measuring a specific type of belief about eating that is believed to maintain binge eating in individuals with eating disorders [16,17]. Based on metacognitive theory [18,19] and the cognitive model of BN [17], the EBQ-18 measures negative beliefs about eating (beliefs that relate to a perception of having no control in relation to food and eating, for example, difficulty with resisting urges to eat and/or stopping eating once an episode of binge eating has started), positive beliefs about eating (beliefs that relate to the perceived benefits of binge eating, such as reducing emotional distress), and permissive beliefs about binge eating (beliefs that one should allow oneself to commence or continue to binge eat). Items for the three EBQ-18 subscales were developed with input from a number of experts in the area of eating disorders and cognitive therapy, for example, clinical psychologists and psychiatrists with training and experience in the assessment and treatment of eating disorders and/or the delivery of cognitive therapy [16,20]. Subscales were refined while using exploratory and confirmatory factor analysis such that the current EBQ-18 contains the six best items from each of the three subscales [15,16,20]. The EBQ-18 can be used to assess for the presence and severity of binge eating related cognitions in both clinical and community samples. The results of a recent study using a large non-clinical sample found that the EBQ-18 demonstrates promising measurement properties, such as good internal consistency, convergent validity, and interpretability with a large non-clinical sample [20]. The factor structure of the EBQ-18 has not yet been verified with a clinical or binge eating sample. An earlier two-subscale version of the EBQ demonstrated evidence of clinical utility and responsiveness to treatment [15], with EBQ scores successfully differentiating individuals with BN or BED from non-clinical control participants, and optimal clinical cut-off scores were identified [21]. In its current three-factor form, the clinical utility of EBQ-18 has yet to be assessed with an eating disorder sample.

According to specific criteria for the assessment of a measure’s methodological quality [22,23], a measure should be able to demonstrate adequacy across a number of important measurement properties, including content validity, internal consistency, criterion validity, construct validity, structural validity, agreement, reliability, responsiveness, floor and ceiling effect, and interpretability. In this paper, we seek to provide a thorough assessment of the EBQ-18’s methodological quality according to the principles described in by Terwee et al. [22] and Mokkink et al. [23] to determine whether the EBQ-18 provides a valid, reliable, and clinically useful measure of eating beliefs within the clinical target population.

Using both a clinical and non-clinical sample, this study aimed to provide a comprehensive assessment of psychometric properties and clinical utility of the EBQ-18 and to identify optimal clinical cut-off scores while using receiver operating characteristics (ROC) curve analysis. We predicted that the confirmatory factor analysis would provide support for the three-factor solution that was identified in the initial exploratory factor analysis [20]: negative beliefs, positive beliefs, and permissive beliefs about eating. In line with previous findings, we hypothesised that the EBQ-18 scores would positively correlate with binge eating frequency, measures of eating disorder symptoms and psychopathology, and other measures of eating-related beliefs, thereby demonstrating convergent validity. We further hypothesised that the EBQ-18 would demonstrate evidence of adequate internal consistency, test-retest reliability over a two-week interval, and sensitivity to treatment following a psychological intervention. It was predicted that the EBQ-18 scores would differ significantly depending on binge eating status, such that those individuals engaging in regular binge eating would score significantly higher on the EBQ-18 and its subscales than those not engaging in binge eating, and those individuals that were diagnosed with a binge-related eating disorder (e.g., BN or BED) would score significantly higher on the EBQ-18 than those individuals with a restrictive eating disorder (e.g., AN). 

## 2. Materials and Methods 

### 2.1. Participants

In total, data from 688 participants were included in this study (63.1% female, mean age = 25.38, standard deviation (SD) = 11.82 years, mean body mass index (BMI) = 24.30, SD = 8.40). This sample consisted of both non-clinical participants (*n* = 498) and treatment-seeking participants (*n* = 190). 

*Non-clinical samples:* First year psychology students from The University of Sydney (*n* = 308) completed the research test battery in exchange for course credit. In addition, participants from the general community (*n* = 190) responded to an advert that was placed on internet-based community classifieds asking for interested volunteers for research.

*Treatment-seeking samples:* Participants were recruited from a number of different metropolitan treatment sites, including hospital-based eating disorder treatment services, private eating disorder treatment services, and hospital-based obesity services. These numbers reflect the total number of consenting participants that were recruited from these sites who provided complete EBQ-18 data and were therefore eligible to be included in the study. Refer to Table 1 for demographic information for these samples.

*Clinical subgroups:* Two subgroups of participants were created based on self-reported binge eating status. Items of the Eating Disorder Examination Questionnaire (EDE-Q) were used to assess self-reported binge eating status of participants (refer to Measures section for more information on this questionnaire). From the total sample of 688,343 participants (52.9%) reported that they had not engaged in any episodes of overeating over the previous 28 days, these 343 participants were grouped for the purposes of providing a non-binge eating comparison sample (referred to as the Non-BE subgroup) for subsequent analyses. Of the total sample of 688,190 participants, (27.9%) self-reported that they engaged in at least four episodes of objective binge eating (objective overeating with a sense of loss of control; OBE) over the previous 28 days and were grouped together for the purpose of providing a binge eating sample (referred to as the BE subgroup). In addition, seven diagnostic subgroups from the treatment-seeking sample were also identified; AN restrictive type (AN-R), AN binge eating/purging type (AN-BP), BN, BED and OSFED atypical AN (OSFED AN), BN of low frequency or limited duration (OSFED BN), and BED of low frequency or limited duration (OSFED BED). Clinical diagnoses were provided by a clinical psychologist or psychiatrist with expertise and training in eating disorder assessment and treatment. Demographics for these subgroups are also reported in Table 1.

### 2.2. Measures

*The Eating Beliefs Questionnaire* (EBQ-18; included in supplementary materials as S1) [20]. The EBQ-18 is an 18-item self-report questionnaire that measures negative, positive, and permissive beliefs about eating and urges to eat in the absence of hunger. Respondents rate how much they agree with 18 statements from 1 (strongly disagree) to 5 (strongly agree). It has demonstrated psychometric properties, as reported in the Introduction. 

*The Eating Disorder Examination Questionnaire* (EDE-Q) [24]. Based on the Eating Disorder Examination interview (EDE; Fairburn, Wilson [25]), the EDE-Q is a self-report questionnaire that assesses of the severity and frequency of eating disorder symptoms (including binge eating) experienced over the 28 days prior. Respondents provide a rating between 0 and 6 for 28 items, where higher scores are indicative of an increased frequency or severity of symptoms. Respondents also provided frequencies for eating disorder behaviours (e.g., overeating, binge eating, purging, driven exercise). The EDE-Q global score provides an indication of the severity of eating disorder symptoms, and four subscales assess severity of dietary restraint, eating concern, weight concern, and shape concern. The EDE-Q has demonstrated evidence of its reliability and validity [26]. Cronbach’s alpha of 0.94 for the EDE-Q global score for the sample in this study.

*Depression Anxiety Stress Scale* (DASS-21) [27]. The DASS-21 is a self-administered screening tool and it was used here to measure symptoms of depression, anxiety, and stress that were experienced over the previous week. The DASS-21 has demonstrated evidence of its reliability and validity [28]. Cronbach’s alpha of 0.95 for the total score in the current study.

*Dutch Eating Behaviour Questionnaire* (DEBQ) [29]. The DEBQ is a self-report scale that measures the presence of particular eating behaviours and attitudes. The current study used the DEBQ emotional eating scale and the DEBQ external eating scale. The third scale of the DEBQ measures restrained eating, as the EDE-Q provides a measure of dietary restraint we did not include the restrained eating scale in our test battery in the interest of reducing repetition and participant burden as much as possible. The emotional eating scale provides a 13-item measure of the influence of emotional or internal cues on eating behaviour, and the external eating scale is a 10-item measure of the influence of environmental or external cues on eating behaviour. Both DEBQ scales have demonstrated adequate internal consistency [29]. Cronbach’s alpha of 0.94 for the emotional eating subscale and Cronbach’s alpha of 0.81 for the external eating subscale were obtained in the current study.

*Eating Disorder Thoughts Questionnaire* (EDTQ) [30]. The EDTQ is a 26-item self-report questionnaire measuring the presence of beliefs about the positive and negative consequences of eating, permissive thoughts and thoughts of no control over eating. The EDTQ has demonstrated evidence of good internal consistency and validity [30]. Cronbach’s alpha of 0.95 was obtained for the whole measure in the current study.

### 2.3. Procedure

*Non-clinical*: Participants completed a battery of questionnaires online using Qualtrics Survey Software. First year university participants (*n* = 308) completed a larger test battery that consisted of all measures that were described in the Materials section. At recruitment, participants could choose to sign up for the standard or two-part version of the study. A sample of 100 participants from the university sample completed the two-part version of the study, which involved completing the EBQ-18 a second time following an interval of two weeks (84% female, mean age = 20.69 years, SD = 7.06 years, mean BMI = 21.63, SD = 2.96). Community participants (*n* = 190) completed a briefer test battery that consisted of demographics items, the EBQ-18, the DASS-21, and the EDE-Q.

*Treatment-seeking:* Participants recruited from clinical settings were offered the opportunity to take part in a questionnaire-based research study by a member of administrative staff. Those who consented to participate then completed a test battery including demographic items, EBQ-18, DASS-21, and the EDE-Q. Diagnoses were recorded by the treating clinician; a clinical psychologist or psychiatrist with expertise and training in the assessment and treatment of eating disorders. The EBQ-18 was administered to clinical participants before and after receiving psychological treatment; in total, thirty-eight participants who received psychological treatment for an eating disorder provided both pre- and post-treatment EBQ-18 scores. 

All the procedures performed in studies involving human participants were in accordance with the ethical standards of the University of Sydney Human Research Ethics Committee (Project Code: 2014_082) and with the 1964 Helsinki Declaration and its later amendments.

### 2.4. Missing Data

For the purposes of the analyses that were reported in this paper, complete EBQ-18 data was required. Where participants were missing one item per EBQ-18 subscale (i.e., 83.3% of items were completed), the missing item was replaced with the mean of the remaining items in that subscale. If more than one item per subscale was missing, then that participant’s data was considered incomplete and excluded from analysis. For other measures, the same approach was taken whereby if one item was missing on a scale or subscale, the missing item was replaced with the mean score, and if more than one item was missing, the data for that measure was considered to be incomplete and excluded from the analysis.

### 2.5. Analyses

Descriptive statistics and analyses of the validity and reliability of the EBQ-18 were conducted using the SPSS v22 program (IBM, New York, NY, USA). Internal consistency was examined with Cronbach’s alpha. Test-retest reliability and construct validity were assessed with Pearson correlations and intraclass correlations (ICC). Between group differences were tested with one-way ANOVAs. Treatment sensitivity was assessed using repeated measures ANOVAs, Cohen’s *d* was used to calculate effect size and cut-off points for clinically significant change were calculated while using ROC Curve analyses. Using a Maximum Likelihood method, the AMOS v12 program [31] was used to conduct the CFA on the EBQ-18 items to evaluate the fit of the data to the three-factor model reflective of the three subscales of negative, positive, and permissive beliefs. The MedCalc program (MedCalc Software, Mariakerke, Belgium) was used to conduct ROC curve analyses to determine optimal clinical cut-off scores and the associated indicators of test performance: area under the curve (AUC), sensitivity, specificity, and positive and negative predictive values.

## 3. Results

### 3.1. Confirmatory Factor Analysis (CFA)

A CFA was conducted in AMOS (IBM, New York, NY, USA) while using a Maximum Likelihood method. The three-factor solution (negative beliefs, positive beliefs and permissive beliefs) was fitted to the data (non–clinical sample, *n* = 498, providing a sample of adequate size with 27.6 participants per item on the scale) and demonstrated good fit [32,33]: *χ*^2^/df = 2.16, Goodness of Fit Index (GFI) = 0.94, Incremental Fit Index (IFI) = 0.96, Tucker Lewis Index (TLo) = 0.96, Comparative Fit Index (CFI) = 0.96, and the Root Mean Square Error of Approximation (RMSEA) = 0.048 with its 90% confidence intervals (CI) 0.041 to 0.056. This supports the findings of previous research [20]. The three-factor model was also fitted to the binge eating subgroup data (BE, *n* = 190, providing a sample with 10.5 participants per item on the scale) and demonstrated adequate fit [32,33]: *χ*^2^/df = 2.37, GFI = 0.85, IFI = 0.89, TLo = 0.87, CFI = 0.89, and the RMSEA = 0.085 with its 90% CI 0.073 to 0.097. Refer to Table 2 for standardised regression weights and communalities of the three-factor solution for the EBQ-18 with a binge eating sample.

### 3.2. Psychometric Properties

The EBQ-18 subscales were found to correlate significantly (*n* = 688): positive and negative, *r* = 0.64, *p* < 0.01*;* negative and permissive, *r* = 0.52, *p* < 0*.*01; and, positive and permissive, *r* = 0.46, *p* < 0.01*.* Across all 688 participants, scores on the EBQ-18 total ranged from the minimum score of 18 and highest score of 85. With regard to the floor and ceiling effects, only 2.6% of participants achieved the lowest possible score, and 0% of participants achieved the highest possible score of 90. Scores on the EBQ-18 subscales ranged from the lowest possible score of 6 (negative scale, 9.2%; positive scale 9.2%; permissive scale 10.2%) to the highest score of 30 (negative scale, 1.2%; positive scale 0.6%) or 29 (permissive scale 0.1%).

### 3.3. Internal Consistency

Cronbach’s alphas were calculated with the full sample (*n* = 688), and for the different sample groups, see Table 3. EBQ-18 subscales were found to have good internal consistency with Cronbach’s alphas ranging between 0.88 and 0.96 for the EBQ-18 total score, Cronbach’s alphas (α) between 0.84 and 0.94 for the negative beliefs scale, Cronbach’s alphas between 0.84 and 0.95 for the Positive Beliefs scale, and Cronbach’s alphas between 0.72 and 0.84 for the Permissive Beliefs scale.

### 3.4. Test-Retest Reliability

Test–retest reliability was calculated for the EBQ-18 total and the three subscales while using a sample of 100 university participants who completed the EBQ-18 a second time after an interval of two weeks. An intraclass correlations (ICC) ≥ 0.70 indicates acceptable reproducibility of a measure [22]. Results showed good test-retest reliability across the EBQ-18 and all three subscales with significant Pearson’s *r* and ICC between Time 1 and Time 2 scores (EBQ-18 Total, *r* = 0.83, ICC = 0.91, *p*’s < 0.001; negative beliefs scale, *r* = 0.74, ICC = 0.85, *p*’s < 0.001; EBQ-18 Positive Beliefs scale, *r* = 0.82, ICC = 0.90, *p*’s < 0.001; Permissive Beliefs scale, *r* = 0.77, ICC = 0.87, *p*’s < 0.001). 

### 3.5. Construct Validity

Convergent validity between EBQ-18 scores and the included measures of specific eating disorder symptoms and related psychopathology was assessed by examining the correlations between the included measures. Correlations between the included measures with the full scale (EBQ Total), Negative Beliefs, and Positive Beliefs scales were all significantly and positively correlated. A number of correlations between the EBQ-18 Permissive Beliefs scale and other measures were not significant, see Table 4. The number of participants included in these correlations differs depending on the total number of the participants that had completed the relevant comparison measures.

### 3.6. Group Differences

Table 5 summarises the mean total scores and subscale scores for the different samples and for subgroups of the participants that are based on self-reported binge eating status, as well as based on formal eating disorder diagnosis as recorded by the participants’ treating clinician.

Results from one-way ANOVAs identified that participants in the BE subgroup scored significantly higher on the EBQ-18 and its subscales than those in the Non-BE subgroup, with moderate-to-large effect sizes (η_p_²); EBQ-18 total score: *F*(1,532) = 280.08, *p* < 0.01, η_p_² = 0.35; Negative Beliefs score: *F*(1,532) = 197.18, *p* < 0.01, η_p_² = 0.27; Positive Beliefs score: *F*(1,532) = 343.51, *p* < 0.01, η_p_² = 0.39; Permissive Beliefs score: *F*(1,532) = 56.51, *p* < 0.01, η_p_² = 0.10. 

In addition, participants with a diagnosis of BN scored significantly higher on the EBQ-18 subscales than participants with a diagnosis of AN-R with large effect sizes (EBQ-18 total score: *F*(1,58) = 155.46, *p* < 0.01, η_p_² = 0.73*;* Negative Beliefs score: *F*(1,58) = 138.71, *p* < 0.01, η_p_² = 0.71; Positive Beliefs score: *F*(1,58) = 81.92, *p* < 0.01, η_p_² = 0.59; Permissive Beliefs score: *F*(1,58) = 62.14, *p* < 0.01, η_p_² = 0.52) and significantly higher than those with a diagnosis of AN-BP with moderate-to-large effect sizes (EBQ-18 total score: *F*(1,44) = 14.42, *p* < 0.01, η_p_² = 0.25*;* Negative Beliefs score: *F*(1,44) = 9.54, *p* < 0.01, η_p_² = 0.18; Positive Beliefs score: *F*(1,44) = 6.36 *p* < 0.05, η_p_² = 0.13; Permissive Beliefs score: *F*(1,44) = 11.52, *p* < 0.01, η_p_² = 0.21).

Participants with a diagnosis of BED also scored significantly higher on the EBQ-18 subscales than the participants with a diagnosis of AN-R, with large effect sizes (EBQ-18 total score: *F*(1,42) = 151.29, *p* < 0.01, η_p_² = 0.79*;* Negative Beliefs score: *F*(1,42) = 106.63, *p* < 0.01, η_p_² = 0.72; Positive Beliefs score: *F*(1,42) = 109.60, *p* < 0.01, η_p_² = 0.73; Permissive Beliefs score: *F*(1,42) = 75.73, *p* < 0.01, η_p_² = 0.65), and significantly higher than those with a diagnosis of AN-BP, with moderate-to-large effect sizes (EBQ-18 total score: *F*(1,28) = 14.21, *p* < 0.01, η_p_² = 0.35*;* Negative Beliefs score: *F*(1,28) = 5.58, *p* < 0.05, η_p_² = 0.17; Positive Beliefs score: *F*(1,28) = 10.32, *p* < 0.01, η_p_² = 0.28; Permissive Beliefs score: *F*(1,28) = 14.67, *p* < 0.01, η_p_² = 0.35).

Participants with AN-BP were found to score significantly higher than those with AN-R for the EBQ total score, *F*(1,33) = 12.91, *p* < 0.01, η_p_² = 0.29, the Negative Beliefs scale, *F*(1,33) = 18.46, *p* < 0.01, η_p_² = 0.37, and the Positive Beliefs scale, *F*(1,33) = 8.10, *p* < 0.01, η_p_² = 0.20, but no significant difference was identified between AN-BP and AN-R on the Permissive Belief scale scores, *p* > 0.05. No significant differences found between BN and BED sample on EBQ-18 total or subscale scores, all *p*’s > 0.05.

### 3.7. Treatment Sensitivity

A total of thirty-eight participants that were seeking treatment for an eating disorder completed the measures both before and after psychological treatment. Ten of these participants had completed an 8 to 10-week hospital-based outpatient binge eating group therapy treatment program (*n* = 5 with a diagnosis of BN and *n* = 5 with a diagnosis of BED). A further twenty-eight of these participants had completed individual psychological treatment at private specialist eating disorder treatment centres (*n* = 5 with a diagnosis of AN-R, *n* = 5 with a diagnosis of AN-BP, *n* = 3 with a diagnosis of BN, *n* = 4 with a diagnosis of BED, *n* = 6 with a diagnosis of OSFED AN, *n* = 1 with a diagnosis of OSFED BN, *n* = 3 with a diagnosis of OSFED BED, and *n* = 1 with a diagnosis of UFED). Treatments, both group and individual, were based on a cognitive-behavioural approach and they were delivered by trained and experienced clinicians. Table 6 summarises the group means, SD, and Cohen’s *d* effect sizes at pre- and post-treatment for the EBQ-18 total score and subscales across the whole treatment sample and the subgroup that includes only those participants with diagnosed eating disorders with binge eating as a core feature (BE).

Results from repeated measures ANOVAs identified that the pre-treatment EBQ-18 scores were significantly higher than post-treatment scores across the total sample of treatment completers (*n* = 38): EBQ-18 total score: *F*(1,37) = 17.48, *p* < 0.01, η_p_² = 0.32; Negative Beliefs score: *F*(1,37) = 15.67, *p* < 0.01, η_p_² = 0.30, Positive Beliefs score: *F*(1,37) = 6.86, *p* < 0.05, η_p_² = 0.16,Permissive Beliefs score: *F*(1,37) = 10.42, *p* < 0.01, η_p_² = 0.22.

We also examined the difference in pre and post-treatment EBQ-18 scores for just those participants who were diagnosed with BED (*n* = 9) or BN (*n* = 8). Results from repeated measures ANOVAs identified that, for the BN/BED subgroup (*n* = 17), pre-treatment EBQ-18 scores were significantly higher than post-treatment scores, with large effect sizes: EBQ-18 total score: *F*(1,16) = 19.60, *p* < 0.01, η_p_² = 0.55; Negative Beliefs score: *F*(1,16) = 18.82, *p* < 0.01, η_p_² = 0.54, Positive Beliefs score: *F*(1,16) = 5.44, *p* < 0.05, η_p_² = 0.25, Permissive Beliefs score: *F*(1,16) = 10.86, *p* < 0.01, η_p_² = 0.40.

In order to assess whether the differences between the pre-treatment scores and post-treatment scores were of clinical significance, we applied the formula from Jacobson and Truax [34] to determine the EBQ-18 score for clinically significant change for each group. We used mean normative data for the EBQ-18 from a non-clinical sample of 883 participants that found a mean of 38.25 and standard deviation of 12.87 for the EBQ-18 total score [20]. Therefore, the score of clinically significant change for both the whole treatment sample, c(Trmt), and the BN/BED subgroup, c(BN/BED), were as follows:

(1) $$c\left(\mathrm{Trmt}\right)=\frac{S0M1+S1M0}{S0+S1}=\frac{12.87\left(49.24\right)+18.73\left(38.25\right)}{12.87+18.73}=\frac{633.72+716.42}{31.6}=42.73$$

(2) $$c\left(\mathrm{BN}/\mathrm{BED}\right)=\frac{S0M1+S1M0}{S0+S1}=\frac{12.87\left(63.47\right)+10.14\left(38.25\right)}{12.87+10.14}=\frac{816.86+387.86}{23.01}=52.36$$

Applying the Jacobson and Truax’s (1991) [34] formula, participants who scored less than 42.73 at post-treatment (and less than 52.36 at post-treatment for those in the BN/BED subgroup) would likely have experienced a clinically significant effect of treatment. Post-treatment means are lower than the identified scores of clinically significant change for the EBQ-18 total score, indicating that meaningful change has occurred as a result of treatment. Of the whole treatment sample, 55.26% scored less than 42.73 at post-treatment, and of the BN/BED subgroup, 64.7% scored less than 52.36 on the EBQ-18 at post-treatment.

### 3.8. Receiver Operating Characteristic (ROC) Curve Analyses

ROC curve analyses were used to identify the optimal clinical cut-off scores for the EBQ-18 and to assess the associated test performance indicators (sensitivity, specificity, and positive and negative predictive values). Here, we report the associated criterion for the Youden-Index, which provides a cut-off score for which both sensitivity and specificity is maximal [35]. For the purposes of these analyses, a non-binge eating control sample of 61 participants from the community sample who self-reported to engage in 0 episodes of objective binge eating in the past 28 days, and a clinical BN/BED sample of 54 participants, including 35 participants with a clinician confirmed diagnosis of BN and 19 participants with a clinician confirmed diagnosis of BED, were utilised. 

Table 7 summarises the cut-off criteria and associated test performance indicators for the EBQ-18 and its subscales for the comparison sample of the combined BN and BED sample versus controls. The cut-off scores that were identified for all of the subscales have significant area under the curve (AUC) values, indicating that the identified cut-off scores perform better than chance in discriminating between clinical cases (those with BN or BED) and non-clinical cases. 

Table 8 summarises the cut-off criteria and associated test performance indicators for the EBQ-18 total score across four different comparison samples (1) BN subgroup versus controls; (2) BED subgroup versus controls; (3) AN-BP versus controls; (4) AN-R versus controls. The cut-off scores identified for BN, BED and AN-R have significant AUC values, indicating that the identified cut-off scores perform better than chance in discriminating between clinical cases and non-clinical cases. The AUC value associated with the identified cut-off score for discriminating those with AN-BP from non-clinical cases was not significant, and therefore this cut-off score may not be a reliable indicator for this group.

## 4. Discussion

This study sought to provide a thorough examination of the validity, reliability, and clinical utility of the EBQ-18 while using both a clinical eating disorder treatment-seeking sample and a non-clinical sample. Overall, the results of this study indicate that the EBQ-18 is a valid, reliable, and clinically useful measure for use in the general population as well as with individuals seeking treatment for binge eating. These findings build upon the previous research that found an earlier, two subscale version of the EBQ to be valid and reliable with clinical and non-clinical samples [15,16,21]. These findings are also consistent with the initial examination of the factor structure, internal consistency, and content validity of the current EBQ-18 with a non-clinical sample [20]. The findings of the present study extend existing research by further assessing the test-retest reliability, comparisons of diagnostic groups, responsiveness to treatment, clinical significance, and identifying clinically useful cut-off scores for the EBQ-18. The results of this study were in keeping with the hypotheses.

### 4.1. Factor Structure

As predicted, the confirmatory factor analysis provided support for the three-factor solution that was identified in the initial exploratory factor analysis [20]: negative beliefs, positive beliefs, and permissive beliefs about eating. The three-factor solution demonstrated good-fit with the non-clinical sample that provided a homogenous sample of adequate size for CFA. When the three-factor solution was fitted to the self-report binge eating sample, we found adequate fit to the data. Although, we would wish for good fit across all of the fit indices, it is important to note that the BE sample was not homogeneous (participants within this sample were recruited across university, community, and treatment-seeking samples) and the sample (*n* = 190) was relatively small with regard to CFA, which finds that fit statistics are more accurate when the sample is larger than *n* = 250 [33]. The CFA with the BE sample also found that one item from the permissive scale did not load as well as other items, “It won’t make a difference if I eat more”, with a standardised regression weight of less than 0.40 and communality of less than 0.20. This low loading item could be impacting the psychometric properties of the Permissive Beliefs scale as a whole. Conceptually, this item is different from other the permissive items that focus more on the theme that binge eating is a pleasant experience that one deserves, and instead this item assesses a sense of allowing oneself to continue a binge once eating has commenced. Despite having the lowest factor loading, this item was retained for its clinical and theoretical value, as well as because this item has demonstrated improved performances in other larger studies assessing the factor structure of the EBQ. Differences across the samples, including the homogeneity of the present sample, might account for this minor difference.

### 4.2. Psychometric Properties

In keeping with our hypotheses and with previous findings, the EBQ-18 and its subscales demonstrated good internal consistency across different sample groups, with all the Cronbach’s alphas over 0.70, thereby meeting the Terwee criteria for adequacy for internal consistency [22]. The EBQ-18 also demonstrated adequacy for agreement, showing good test-retest reliability over two weeks with a sample of 100 university students with strong, significant correlations between Time 1 and Time 2 for the EBQ-18 and its three subscales.

Adequate content validity was also demonstrated with the EBQ-18 Total, Negative Beliefs and Positive Belief subscale scores positively and significantly correlated with the measures of binge eating frequency and other eating disorder symptoms (EDE-Q), other eating disorder measures (DEBQ; EDTQ), and a measure of depression, anxiety, and stress (DASS-21). The Permissive Beliefs scale did not correlate with EDE-Q Global Score, Eating Concerns, Shape Concerns, and Weight Concerns subscales. However, the Permissive Beliefs scale did negatively correlate with the EDE-Q Restraint subscale. As the EDE-Q Restraint subscale measures the individual’s recent attempts at restrictive eating practices, such as dieting and fasting, it is unsurprising to find that it is negatively correlated with the Permissive Beliefs scale, which measures beliefs about allowing for oneself to engage in binge eating. Furthermore, this interesting finding differs from the correlations between the EBQ-18 Permissive Beliefs scale and the EDE-Q subscale in a large non-clinical sample in Burton & Abbott (2018) [20], who found small, significant correlations between the EDE-Q Global Score, Eating Concerns, Shape Concerns, and Weight Concerns subscales and the Permissive Beliefs scale, and a non-significant negative correlation between the Restraint subscale and the Permissive Beliefs scale. This difference in the results can be accounted for by the difference in the samples used in the two different studies with the present study, including a clinical, eating disordered sample and overall a higher proportion of the whole sample self-reporting to be engaging in regular binge eating (28% in the present study compared to 19% in Burton & Abbott, 2018) [20]. It is important to note that, while the correlations between the EBQ-18 and the included measures were significant and in the expected direction, they are relatively small correlations.

### 4.3. Clinical Utility

As predicted, the EBQ-18 scores were found to differ significantly depending on binge eating status, such that those individuals engaging in regular binge eating scored significantly higher on the EBQ-18 than those not engaging in binge eating, and that those individuals diagnosed with BN, BED, or AN-BP scored significantly higher on the EBQ-18 than those individuals with AN-R. There was no significant difference in EBQ-18 scores between those with a diagnosis of BN and those with BED, further supporting the role of metacognitive beliefs in theoretical models of BN, BED, and transdiagnostic models [17,20].

Furthermore, adequacy for responsiveness to treatment was demonstrated with a significant reduction in EBQ-18 scores following a psychological intervention for eating disorders. The effect of treatment on EBQ-18 scores was especially strong (large Cohen’s *d*) for those with BN or BED. The treatment effect was also found to indicate clinically significant, or meaningful, change with post-treatment means lower than the cut-off for clinically significant change calculated using the formula from Jacobson and Truax [34]. Using ROC Curve Analysis, optimal cut-off scores for the EBQ-18 and its subscales were identified. With significant AUC values, the cut-off scores that were identified can reliably be used to discriminate clinical cases, those with BED and BN, from non-clinical cases [36,37]. That is, respondents scoring higher than 45 on the EBQ-18 total score can be considered to be experiencing a clinically significant level of eating disorder related beliefs about eating. However, it is important to note that the results of the ROC curve analysis did not provide support for the use of the identified cut-off score to be used to discriminate AN-BP cases from non-clinical cases. Interestingly, very low scores on the EBQ-18 can be informative too, with results indicating that a score of 23 or less could discriminate the cases of restrictive type AN from non-clinical cases. The EBQ-18 could therefore provide an informative addition to test batteries assessing for the presence or nature of disordered eating beliefs and behaviours. One potential application of this would be for the EBQ-18 to be administered to candidates for bariatric surgery to assess for the presence of eating disorder related cognitions. Furthermore, the EBQ-18 can be used in research examining the predictors of successful outcomes for obesity treatments, such as bariatric surgery or cognitive and behavioural interventions.

### 4.4. Limitations and Future Direction

One limitation of this study is that the evaluation of responsiveness to treatment used a mixed sample, including both a group treatment for binge eating and individual treatment for a range of eating disorder presentations from a number of different specialised private and public eating disorder treatment clinics. Furthermore, the treatment used did not explicitly address or challenge the specific unhelpful beliefs about eating that are measured by the EBQ-18. Future research should compare change in eating beliefs over course of treatment between treatment-as-usual (CBT) and a metacognitive therapy approach, such as that outlined in Cooper, Todd, and Wells [38], which explicitly targets the underlying beliefs about eating thought to maintain binge eating. A further potential limitation is the relatively small size of the sample for the treatment sensitivity analyses (*n* = 38), this relatively small *n* was due to participant attrition following initial assessment and also due to participants not completing the follow-up surveys after completing treatment. However, this clinical sample size is comparable to the clinical sample sizes that were used in eating disorder research [15], and we did not find that this sample size limited our results, obtaining significant findings with regard to the EBQ-18’s sensitivity to treatment.

Furthermore, while the EBQ-18 and subscales demonstrated good test-retest reliability over a two-week period, future research should also assess the medium and longer-term temporal stability of the EBQ-18. Future studies could also assess the use of the EBQ-18 in other relevant sample groups that were not included in this study, such as in individuals who are overeating without a sense of loss of control and those who are engaging in subjective binge eating episodes (sense of loss of control over eating, but without objective overeating).

## 5. Conclusions

Overall, this paper provides new information on the factor structure, validity, reliability, and clinical utility of the EBQ-18 across clinical and non-clinical samples. Notably, the results of this paper add to the existing literature on the relevance of the three types of eating beliefs that were measured by this questionnaire: negative, positive and permissive beliefs about eating. Endorsement of these beliefs have been found to be significantly higher in individuals engaging in binge eating than those who do not report binge eating, as well as endorsement of these beliefs being found to be significantly higher in those individuals experiencing BN and BED as compared to other eating disorder and control groups. We have also found that these beliefs reduce significantly as a result of psychological treatment.

Given the increasing prevalence and seriousness of the associated co-morbid conditions of binge eating, including obesity, other physical and mental health conditions, and poorer quality of life, it is important that clinicians and researchers have access to good quality, informative and easy-to-administer assessment tools that can help to inform clinical formulation and to monitor change over the course of treatment. The EBQ-18 is a valid, reliable, and clinically useful brief self-report assessment of the presence of three types of specific unhelpful beliefs (negative, positive, and permissive beliefs about eating), which are considered to maintain binge eating in individuals with disordered eating. The EBQ-18 demonstrated evidence of good psychometric properties within a clinical treatment-seeking sample, a sample self-reporting to be engaging in regular binge eating, and a non-clinical sample. 

## Figures and Tables

**Table 1:** Demographic Information for participant samples.

Sample	*n*	Mean Age (SD)	Mean BMI (SD)	% Female	Mean OBEs in Past 28 Days (SD)
*Non-clinical samples:*					
First Year University Students	308	19.66 (4.54)	22.08 (3.81)	80.8	2.18 (4.32)
General Community	190	31.22 (12.83)	25.10 (6.08)	62.6	3.19 (6.21)
*Treatment-seeking samples:*					
Hospital-based Obesity Treatment Service	29	50.97 (14.23)	46.53 (12.56)	55.2	2.32 (3.29)
Hospital-based Outpatient Eating Disorder Treatment Service	19	30.29 (8.86)	26.59 (13.24)	100	7.06 (9.90)
Hospital-based Outpatient Binge Eating Group Treatment Program	31	28.00 (11.72)	25.85 (5.68)	100	20.43 (18.65)
Private Eating Disorder Treatment Services	111	23.57 (9.92)	23.20 (11.20)	91.9	7.15 (10.20)
*Subgroup based on self-reported binge eating status:*					
Non-BE	343	25.30 (12.62)	23.53 (7.40)	73.2	0
BE	190	25.39 (10.38)	24.98 (7.97)	87.3	13.14 (11.00)
*Eating Disorder Subgroups:*					
AN-R	24	22.00 (9.27)	18.82 (3.67)	91.7	1.70 (4.97)
AN-BP	10	21.20 (4.94)	17.39 (3.07)	100	12.00 (14.52)
BN	35	25.81 (6.96)	25.06 (7.94)	97.1	18.03 (14.28)
BED	19	31.85 (13.69)	34.57 (13.41)	89.5	19.24 (19.10)
OSFED AN	12	24.25 (10.82)	21.76 (4.38)	100	5.00 (6.15)
OSFED BN	4	23.00 (5.77)	25.42 (3.87)	100	6.50 (5.97)
OSFED BED	4	34.67 (4.16)	25.56 (5.03)	75.0	19.75 (21.48)

SD = standard deviation, BMI = body mass index, OBE = objective binge eating episodes, Non-BE = participants who self-reported 0 incidents of objective binge episodes in 28 days prior to completing the test battery; BE = participants who self-reported engaging in 4 or more incidents of objective binge eating episodes in the 28 days prior to completing the test battery, AN-R = anorexia nervosa restrictive type, AN-BP = anorexia nervosa binge/purge type, BN = bulimia nervosa, BED = binge eating disorder, OSFED = other specified feeding or eating disorder.

**Table 2:** Results of a Confirmatory Factor Analysis of the Eating Beliefs Questionnaire (EBQ-18) with the binge eating sample (*n* = 190) Standardised Regression Weights & Communalities.

EBQ-18 Items	F1: Negative Beliefs	F2: Positive Beliefs	F3: Permissive Beliefs	Communality (h^2^)
I’m not able to control my urges to eat	0.71			0.51
Once I start eating I can’t stop	0.78			0.61
I have no willpower in relation to food	0.79			0.63
I can’t control my eating because I am weak	0.82			0.67
If I don’t control myself I would never stop eating	0.62			0.38
There is nothing I can do to stop eating	0.66			0.44
Eating means I don’t have to think about negative things		0.70		0.49
Eating helps to control my emotions		0.77		0.59
Eating keeps my feelings at a tolerable level		0.76		0.57
Eating helps me to cope with negative thoughts		0.83		0.69
Eating helps me cope with negative feelings		0.85		0.73
Eating is my best way of coping with unwanted feelings		0.73		0.54
Bingeing is something that I can have for myself			0.48	0.24
I deserve to have a pleasure like binge eating			0.61	0.37
It’s okay to have the nice experience of binge eating			0.62	0.38
Bingeing allows me to have something nice for myself			0.82	0.68
It won’t make a difference if I eat more			0.26	0.07
I like to binge			0.57	0.33

F1 = Factor 1, F2 = Factor 2, F3 = Factor 3.

**Table 3:** Internal Consistency of EBQ Scales across Different Sample Groups.

Sample	EBQ-18 Total (α)	Negative Beliefs Scale (α)	Positive Beliefs Scale (α)	Permissive Beliefs Scale (α)
Full Sample (*n* = 688)	0.92	0.90	0.91	0.83
Sample Groups:				
Community Sample (*n* = 190)	0.90	0.88	0.90	0.83
University Sample (*n* = 308)	0.90	0.84	0.88	0.84
Binge Eating Group Sample (*n* = 31)	0.88	0.88	0.92	0.74
Obesity Sample (*n* = 29)	0.94	0.87	0.85	0.82
Hospital Eating Disorder Treatment Sample (*n* = 19)	0.92	0.84	0.84	0.72
Private Eating Disorder Treatment Sample (*n* = 111)	0.96	0.94	0.95	0.81

**Table 4:** Correlations between EBQ-18 Scores and other symptom and cognitive measures.

Scale	EBQ-18 Total Score	Negative Beliefs Scale	Positive Beliefs Scale	Permissive Beliefs Scale
DASS-21 Depression (*n* = 645)	0.24 **	0.22 **	0.24 **	0.14 **
DASS-21 Anxiety (*n* = 645)	0.16 **	0.15 **	0.12 **	0.15 **
DASS-21 Stress (*n* = 645)	0.27 **	0.26 **	0.25 **	0.14 **
EDE-Q-Objective Binge Episodes (OBE) (*n* = 650)	0.48 **	0.45 **	0.50 **	0.25 **
EDE-Q Global Score (*n* = 650)	0.30 **	0.33 **	0.42 **	−0.03
EDE-Q Restraint (*n* = 650)	0.12 **	0.17 **	0.23 **	−0.14 **
EDE-Q Eating Concern (*n* = 650)	0.36 **	0.36 **	0.47 **	0.05
EDE-Q Shape Concern (*n* = 650)	0.33 **	0.35 **	0.41 **	0.02
EDE-Q Weight Concern (*n* = 650)	0.29 **	0.32 **	0.41 **	−0.05
DEBQ Emotional Eating (*n* = 201)	0.62 **	0.62 **	0.52 **	0.34 **
DEBQ External Eating (*n* = 201)	0.39 **	0.31 **	0.43 **	0.21 **
EDTQ–Negative Thoughts (*n* = 201)	0.42 **	0.34 **	0.55 **	0.14 *
EDTQ–Positive Thoughts (*n* = 201)	0.64 **	0.62 **	0.52 **	0.36 **
EDTQ–Permissive Thoughts (*n* = 201)	0.60 **	0.53 **	0.43 **	0.51 **

** = *p* < 0.01 (two-tailed), * = *p* < 0.05 (two-tailed), EBQ-18 = Eating Beliefs Questionnaire 18-item version, DASS-21 = Depression Anxiety Stress Scales 21-item version, EDE-Q = Eating Disorders Examination Questionnaire, DEBQ = Dutch Eating Behaviours Questionnaire, EDTQ = Eating Disorders Thoughts Questionnaire.

**Table 5:** EBQ-18 Subscale means and SDs for different sample groups.

Sample	Total EBQ-18 Score Mean (SD)	Negative Beliefs Mean (SD)	Positive Beliefs Mean (SD)	Permissive Beliefs Mean (SD)
University Sample (*n* = 308)	41.50 (11.80)	14.57 (5.27)	12.73 (4.42)	14.20 (4.92)
Community Sample (*n* = 190)	42.21 (13.14)	14.71 (6.07)	13.20 (5.42)	14.29 (5.38)
Hospital-based Obesity Treatment-seeking Sample (*n* = 29)	42.76 (17.15)	14.76 (6.13)	14.83 (6.31)	13.17 (5.29)
Hospital-based Outpatient Eating Disorder Treatment-seeking Sample (*n* = 19)	42.05 (15.97)	14.79 (6.33)	16.05 (6.93)	11.21 (4.45)
Hospital-based Outpatient Binge Eating Group Treatment-seeking Sample (*n* = 31)	63.06 (12.15)	22.65 (4.88)	20.74 (5.33)	19.67 (4.64)
Private Eating Disorder Treatment-seeking Sample (*n* = 111)	42.52 (18.59)	16.26 (7.65)	14.90 (7.68)	11.36 (5.17)
*Binge Eating Subgroups:*				
Non-BE (*n* = 343)	35.90 (11.29)	12.57 (4.86)	10.85 (3.89)	12.48 (5.21)
BE (*n* = 190)	53.89 (12.89)	19.32 (6.04)	18.62 (5.74)	15.96 (4.93)
*Eating Disorder Subgroups:*				
AN-R (*n* = 24)	26.86 (9.57)	9.17 (3.85)	9.42 (4.18)	8.29 (3.26)
AN-BP (*n* = 10)	44.20 (18.70)	17.30 (7.21)	15.60 (8.59)	11.30 (5.54)
BN (*n* = 35)	62.29 (11.43)	23.43 (5.00)	21.17 (5.33)	17.69 (5.17)
BED (*n* = 19)	64.42 (10.39)	22.47 (4.60)	23.37 (4.54)	18.58 (4.49)
OSFED AN (*n* = 12)	35.25 (11.04)	13.50 (4.62)	11.50 (4.83)	10.25 (3.67)
OSFED BN (*n* = 4)	54.50 (9.18)	18.75 (3.77)	21.00 (3.56)	14.75 (4.19)
OSFED BED (*n* = 4)	50.25 (15.06)	21.25 (7.41)	15.50 (4.36)	13.50 (4.65)

EBQ-18 = Eating Beliefs Questionnaire 18-item version, SD = standard deviation, Non-BE = participants who self-reported 0 incidents of objective binge episodes in 28 days prior to completing the test battery; BE = participants who self-reported engaging in 4 or more incidents of objective binge eating episodes in the 28 days prior to completing the test battery, AN-R = anorexia nervosa restrictive type, AN-BP = anorexia nervosa binge/purge type, BN = bulimia nervosa, BED = binge eating disorder, OSFED = other specified feeding or eating disorder.

**Table 6:** Means, SDs and Cohen’s d effect sizes for EBQ-18 scores at pre and post treatment.

Sample	EBQ-18 Total Score Mean (SD)			Negative Beliefs Mean (SD)			Positive Beliefs Mean (SD)			Permissive Beliefs Mean (SD)		
	Pre	Post	*d*	Pre	Post	*d*	Pre	Post	*d*	Pre	Post	*d*
All Treatment (*n* = 38)	49.24 (18.73)	40.11 (14.76)	0.54	18.21 (7.57)	14.18 (6.27)	0.58	17.50 (7.59)	14.82 (6.46)	0.38	13.53 (5.89)	11.16 (4.43)	0.45
BN/BED (*n* = 17)	63.47 (10.14)	51.30 (9.48)	1.24	22.88 (5.11)	17.94 (5.30)	0.95	22.41 (5.29)	19.53 (4.17)	0.60	18.17 (4.73)	13.94 (3.47)	1.02

EBQ-18 = Eating Beliefs Questionnaire 18-item version, SD = standard deviation, BN/BED = Combined bulimia nervosa and binge eating disorder subgroup sample, *d* = Cohen’s *d* effect size.

**Table 7:** Results from ROC Curve Analyses for EBQ Total and subscale scores.

Sample	EBQ-18 Scale	Cut-Off Score	Sensitivity (95% CI)	Specificity (95% CI)	PPV (95% CI)	NPV (95% CI)	AUC (95% CI)
BN/BED (*n* = 54) vs. Control (*n* = 61)	EBQ-18 Total Score	>45	92.59 (82.1–97.9)	93.44 (84.1–98.2)	92.6 (82.9–97.0)	93.4 (84.7–97.3)	0.98 ** (0.93–0.99)
	Negative Beliefs Scale	>16	88.89 (77.4–95.8)	88.52 (77.8–95.3)	87.3 (77.2–93.3)	90.0 (80.8–95.1)	0.95 ** (0.89–0.98)
	Positive Beliefs Scale	>14	90.74 (79.7–96.9)	93.44 (84.1–98.2)	92.5 (82.6–96.9)	91.9 (83.1–96.3)	0.97 ** (0.92–0.99)
	Permissive Beliefs Scale	>13	81.48 (68.6–90.7)	70.49 (57.4–81.5)	71.0 (61.9–78.6)	81.1 (70.6–88.5)	0.83 ** (0.75–0.89)

EBQ-18 = Eating Beliefs Questionnaire 18-item version, CI = Confidence Intervals, PPV = Positive predictive value, NPV = Negative predictive value, AUC = Area under the curve, BN/BED = Combined bulimia nervosa and binge eating disorder subgroup sample, ** = *p* < 0.001.

**Table 8:** Results from receiver operating characteristics (ROC) Curve Analyses for EBQ-18 total score across different diagnostic groups.

Sample	Cut-off Score	Sensitivity (95% CI)	Specificity (95% CI)	PPV (95% CI)	NPV (95% CI)	AUC (95% CI)
BN (*n* =35) vs. Control (*n* = 61)	>50	85.71 (69.4–95.2)	96.72 (88.7–99.6)	93.7 (79.2–98.2)	92.2 (84.0–96.4)	0.97 ** (0.92–0.99)
BED (*n* =19) vs. Control (*n* = 61)	>46	100.00 (82.4–100.0)	95.08 (86.3–99.0)	86.4 (67.8–95.0)	100.00	0.99 ** (0.93–1.00)
AN-BP (*n* = 10) vs. Control (*n* = 61)	>46	40.00 (12.2–73.8)	95.08 (86.3–99.0)	57.1 (25.9–83.6)	90.6 (85.3–94.1)	0.69 (0.57–0.79)
AN-R (*n* = 24) vs. Control (*n* = 61)	≤23	58.33 (36.6–77.9)	78.69 (66.3–88.1)	51.9 (37.4–66.0)	82.8 (74.6–88.7)	0.68 ** (0.57–0.78)

EBQ-18 = Eating Beliefs Questionnaire 18-item version, CI = Confidence Intervals, PPV = Positive predictive value, NPV = Negative predictive value, AUC = Area under the curve, BN = bulimia nervosa, BED = binge eating disorder, AN-BP = anorexia nervosa binge/purge type, AN-R = anorexia nervosa restrictive type, ** = *p* < 0.001.

## Supplementary Materials

The following are available online at http://www.mdpi.com/2072-6643/10/9/1306/s1, S1: EBQ-18—Eating Beliefs Questionnaire.
