# Supplementary material for: Beliefs about Binge Eating: Psychometric Properties of the Eating Beliefs Questionnaire (EBQ-18) in Eating Disorder, Obese, and Community Samples
Source: Nutrients. 2018 Sep 14;10(9):1306. doi: 10.3390/nu10091306 (PMC6165353; doi:10.3390/nu10091306)
Supplement: Supplementary file 1 [file nutrients-10-01306-s001.pdf]

S1. EBQ-18 – Eating Beliefs Questionnaire

Listed below are a number of beliefs people have expressed in relation to food and eating. Circle the number on the scale below to indicate how much you agree with each statement. Please respond to all the items. There are no right or wrong answers.

The items below refer to eating, or urges to eat, that occur when you are **NOT HUNGRY**. The items do not relate to eating, or urges to eat, that occur in response to normal hunger signals or appetite.

**1**	**2**	**3**	**4**	**5**
**Strongly disagree**	**Disagree**	**Unsure**	**Agree**	**Strongly agree**

1.	I'm not able to control my urges to eat	1	2	3	4	5
2.	Eating means I don't have to think about negative things	1	2	3	4	5
3.	Bingeing is something that I can have for myself	1	2	3	4	5
4.	Once I start eating I can't stop	1	2	3	4	5
5.	Eating helps to control my emotions	1	2	3	4	5
6.	I deserve to have a pleasure like binge eating	1	2	3	4	5
7.	I have no willpower in relation to food	1	2	3	4	5
8.	Eating keeps my feelings at a tolerable level	1	2	3	4	5
9.	It's okay to have the nice experience of binge eating	1	2	3	4	5
10.	I can't control my eating	1	2	3	4	5
11.	Eating helps me to cope with negative thoughts	1	2	3	4	5
12.	Bingeing allows me to have something nice for myself	1	2	3	4	5
13.	If I don't control myself I would never stop eating	1	2	3	4	5
14.	Eating helps me to cope with negative feelings	1	2	3	4	5
15.	It won't make a difference if I eat more	1	2	3	4	5
16.	There is nothing I can do to stop eating	1	2	3	4	5
17.	Eating is my best way to cope with unwanted feelings	1	2	3	4	5
18.	I like to binge	1	2	3	4	5

EBQ-18 Scoring Information:

Eating Beliefs Total Score: All items
Negative Beliefs Scale: 1, 4, 7, 10, 13, 16
Positive Beliefs Scale: 2, 5, 8, 11, 14, 17
Permissive Beliefs Scale: 3, 6, 9, 12, 15, 18

Interpretation: Higher scores indicate increased severity in eating psychopathology
